# Parasite Transmission in Social Interacting Hosts: Monogenean Epidemics in Guppies

**DOI:** 10.1371/journal.pone.0022634

**Published:** 2011-08-29

**Authors:** Mirelle B. Johnson, Kevin D. Lafferty, Cock van Oosterhout, Joanne Cable

**Affiliations:** 1 School of Biosciences, Cardiff University, Cardiff, United Kingdom; 2 Western Ecological Research Center, U.S. Geological Survey, Marine Science Institute, University of California Santa Barbara, Santa Barbara, California, United States of America; 3 School of Environmental Sciences, University of East Anglia, Norwich, United Kingdom; University of California, Berkeley, United States of America

## Abstract

**Background:**

Infection incidence increases with the average number of contacts between susceptible and infected individuals. Contact rates are normally assumed to increase linearly with host density. However, social species seek out each other at low density and saturate their contact rates at high densities. Although predicting epidemic behaviour requires knowing how contact rates scale with host density, few empirical studies have investigated the effect of host density. Also, most theory assumes each host has an equal probability of transmitting parasites, even though individual parasite load and infection duration can vary. To our knowledge, the relative importance of characteristics of the primary infected host vs. the susceptible population has never been tested experimentally.

**Methodology/Principal Findings:**

Here, we examine epidemics using a common ectoparasite, *Gyrodactylus turnbulli* infecting its guppy host (*Poecilia reticulata*). Hosts were maintained at different densities (3, 6, 12 and 24 fish in 40 L aquaria), and we monitored gyrodactylids both at a population and individual host level. Although parasite population size increased with host density, the probability of an epidemic did not. Epidemics were more likely when the primary infected fish had a high mean intensity and duration of infection. Epidemics only occurred if the primary infected host experienced more than 23 worm days. Female guppies contracted infections sooner than males, probably because females have a higher propensity for shoaling.

**Conclusions/Significance:**

These findings suggest that in social hosts like guppies, the frequency of social contact largely governs disease epidemics independent of host density.

## Introduction

What drives the probability of epidemics? Characteristics of the parasite, the primary infected host, and the susceptible population might affect whether an epidemic occurs. When a parasite's reproductive ratio (*Ro*) is greater than unity, it can spread through a host population. For directly transmitted parasites, *Ro* is the product of transmission efficiency (*β*), contact rate (*c*) and the duration (*d*) that an infected host is contagious [1]. Knowledge of the factors that affect transmission efficiency, contact rate and the duration of infection, are therefore essential for understanding most infectious disease transmission.

Two modes of transmission are commonly recognised. With density-dependent transmission, the rate of contact is assumed to increase directly with the density of the population [e.g. 2]. Alternatively, when the rate of contact is constant irrespective of population density, transmission is dependent on the relative frequency of susceptible hosts in the population [e.g. 3]. For example, shoaling fish might have a contact rate that is not greatly affected by the density of the population. In such cases, parasite transmission should be governed by frequency-dependent, rather than by density-dependent factors. Evidence exists for both modes of transmission and these modes of transmission have different dynamics [4]. In frequency-dependent transmission models, infected hosts contact other individuals even when density is low, allowing a parasite to invade a low-density host population [5]. Also, whereas host-specific parasites with density-dependent transmission will not generally drive their hosts extinct, parasites with frequency-dependent transmission can do so [6]. Some transmission functions capture the effects of both density and frequency dependence. For instance, at sufficiently high densities, contact rates may saturate, leading to frequency-dependent transmission [5], [7].

Several empirical studies support the assumption that aspects of transmission increase with host density [8]–[10]. For instance, strongylid nematodes are more abundant in abundant mammal hosts [11], and bacterial epidemics are more frequent at sites with high densities of sea urchin hosts [12]. Furthermore, the spread of *Bacillus thuringiensis* [13] and granulosos virus [14] increase strongly with the density of susceptible meal moths. Alternatively, the probability of transmission may be only weakly associated with density [15], [16], such as when sexual interactions are the primary determinants of contact rates among individuals [5]. In those cases, frequency-dependence is a more appropriate transmission model than density-dependence [3].

Characteristics of the primary infected host can greatly affect whether an epidemic occurs. Typhoid Mary, Patient zero (HIV), and various patients in the SARS epidemic were singled out for the unusually large number of secondary infections they were linked to. Very social hosts that are very infectious for a very long time will be more likely to initiate an epidemic and have been termed “super spreaders” [17]. However, the relative importance of the primary infected host vs. the susceptible population has not been investigated experimentally.

Guppy (*Poecilia reticulata*) parasites may experience frequency-dependent transmission given that their hosts have promiscuous sexual behaviour and tend to shoal. Close contact while shoaling may facilitate parasite transmission between hosts [18]–[19]. Females tend to shoal more than males [20], and individual male guppies regularly switch between shoals whilst searching for mating opportunities [21]. Guppies exposed to higher predation pressures tend to shoal more than those in low-risk habitats [e.g. 22], and, in addition, the incidence of sneaky mating is higher in such high-predation habitats [23].

Common parasites of both wild and ornamental guppies are *Gyrodactylus turnbulli* and *G. bullatarudis* [24]. These gyrodactylids are small (<1 mm in length) ectoparasites of fish that can directly transmit from one host to another during host contact (reviewed in [25]). They better meet the assumptions of micro- rather than macroparasite models (transmission through contact of uninfected with infected individuals instead of via parasite eggs, *in situ* reproduction on the host instead of production of a specific free-living transmission stage, and epidemic rather than endemic population growth). Their short generation time and viviparous reproduction can lead to explosive population growth, varying from a few to thousands of worms per fish, but fish can develop immunity to worms over time [e.g. 26]. Although microparasite models assume that all infected hosts have the same potential for transmission, in reality, hosts infected by microparasites often differ in transmission potential [27]. One advantage of the guppy-*Gyrodactylus* system is that it is possible to count the parasite burden of gyrodactylids on each guppy over time. High parasite burdens are typically lethal to the host [24], [28], [29]. In Trinidad, mark-release-recapture studies have shown that gyrodactylid infections can significantly reduce the recapture rate (survival) of guppies, particularly in spate conditions during the wet season rains [29].

Using the guppy–*G. turnbulli* host–parasite system, we investigated the long-term sustainability of a parasite suprapopulation (total number of parasites in the host population, [30]) at different host densities and examined how host density influenced gyrodactylid transmission. To illustrate our predictions, we consider a simple model for the probability of an epidemic, E, given a primary infected host as p(E) = 1−1/c*βd* [1]. If contact rates (*c)* are density-dependent, epidemics should increase in likelihood at higher fish densities. Alternatively, if contacts are based on a relatively constant rate of social interactions e.g. due to shoaling (i.e. frequency-dependent transmission), the likelihood and intensity of epidemics should not increase with host density (so long as duration and transmission per contact do not increase with density). In this case, female guppies are predicted to contract an infection earlier in the epidemic than males, because females tend to shoal more than males [20]. We also predicted that the likelihood of an epidemic would increase with the duration (d) of infection in the primary infected fish. Finally, we expected that a higher intensity of infection on the primary infected fish would increase the chance of an epidemic because high intensity would likely increase transmission efficiency *β*. As per the equation above, we approximated the product of duration and transmission efficiency (*βd)* as the worm days (duration of infection multiplied by the mean number of worms during an infection) experienced by the primary infected fish.

## Materials and Methods

### Ethics statement

All animal work was approved by UK Home Office regulations (PPL 30/2357).

### Fish populations and their maintenance

The guppies used in this study were F2/F3 generation ornamental fish obtained from an aquarium wholesale supplier. All fish were adults, standardized for size and were naïve (i.e., bred in parasite-free conditions). They were individually marked with one or two visible elastomer implants (VIE, Northwest Marine Technology Inc.). Fish were then randomly assigned to one of four density treatments: three guppies (10 replicates), six guppies (9 replicates), 12 guppies (10 replicates) and 24 guppies (2 replicates; Table 1). All treatments contained a ratio of 2 females to 1 male. These densities and sex ratios are comparable to levels in the wild. In Trinidadian guppy populations, Croft et al. [21] estimated that contact occurred between a focal guppy and a conspecific every 14 s at an average density of 12 guppies m^−2^ (approximately equivalent to 12 guppies in 100 L of water with a depth of 10 cm). Fish were allowed to acclimate to each other for approximately one week prior to infection. Experimental aquaria (61 cm length×30 cm width×38.5 cm height) in the current study were filled with 40 L of dechlorinated tap water. Each tank also contained a box filter and artificial plants and black plastic flowerpots for refugia. Replicate tanks were randomly arranged with a 12∶12 h light∶dark photoperiod at 25±0.5°C. Fish were fed twice daily with Aquarian® fish flakes and weekly with frozen bloodworm (*Tubifex* sp.) and/or live *Artemia*. Nine mortality controls were monitored every day using sham-infected fish that had no contact with parasites (3 each of the 3-fish treatment and 6-fish treatment, 2 replicates of the 12-fish treatment and a single replicate of the 24-fish treatment).

**Table 1:** Tank level descriptions of epidemics.

Treatment	n	% epidemics	Days to infection of all fish	Days to peak	Max	Days to parasite extinction
3-fish	10	70	8 (7–14)	9.5	55.8	23.3 (4–52)
6-fish	9	88.9	14 (14)	9	29.1	32.7 (14–62)
12-fish	10	70	35 (14–56)	12	39.4	35.7+(7–77+)
24-fish	2	100	20 (16–24)	26	574.5	62+(50–74+)

Percentage of replicates in which transmission occurred from the primary infected to exposed fish, time for all hosts in a replicate to become infected (and range), average time to peak (suprapopulation) parasite load, mean maximum parasite burden per tank and extinction day (and range) across different tanks (n) per treatment. + indicates the two longest running replicates; frequent screening ceased on day 74 or 77, thereafter screened every 2 weeks, but both of these tanks went extinct by day 98.

### Experimental infections

Primary infected fish were inoculated with the *Gt3* strain of *Gyrodactylus turnbulli* used in several of our previous studies [e.g.], [ 31–32]. Briefly, a single, guppy female from each treatment was anaesthetized with 0.02% MS222 and placed in a Petri dish containing dechlorinated water together with an anaesthetized infected (donor) fish. Their tails were brought into contact, under a stereo-microscope with fibre-optic epi-illumination to allow the transfer of four individual gyrodactylids. The inoculation of the primary infected fish was defined as Day 0, and, immediately after inoculation, fish were returned to their test aquaria. The following day (Day 1), the primary infected fish was monitored to ensure that at least one parasite was present. Any primary infected that had lost all worms by day 1 was re-infected with an additional four parasites, and the time reset to Day 0. Parasite infections were screened on all fish at regular intervals (either every one, two or seven days). There was no significant effect of handling frequency on the mortality of the fish host (Fisher Exact test: P = 0.060). Furthermore, there was no consistent trend in differences in parasite loads associated with observation frequency suggesting that variation in parasite load is not dependant on screening intervals. In one replicate from the 12-fish and 24-fish treatments, the parasite suprapopulation survived for over 2 months and frequent screenings were terminated after 74 and 77 days, respectively. We had planned to screen these replicates every two weeks thereafter, but parasite extinction occurred in both replicates at the first two-week screen (see Table 1). During each screening, all fish from a replicate tank were gently scooped up into individual 1 L containers (without the use of a net, to avoid dislodging the ectoparasites) and then anaesthetized. Each fish was transferred to a small Petri dish containing dechlorinated water and examined under a stereomicroscope. The number and position of worms on each fish was recorded. A parasite suprapopulation was considered to be extinct in a replicate treatment when no parasite was found on any fish after three consecutive screenings [33]. Dead fish were left for 24 h within the tank (to allow transfer of parasites) and then replaced with an uninfected guppy [as in 34] in order to maintain a constant host density. However, such replacement fish were excluded from subsequent analyses (e.g., in terms of estimating prevalence or mean abundance).

### Statistical analyses

By tracking individual fish, we were able to plot the course of an epidemic at the individual level (parasite infrapopulations [30]) as well as the suprapopulation (tank) level. As fish were not monitored daily in all tanks, we estimated the day of first infection and termination of infection with a linear interpolation of abundance (rounded to the nearest day). Subtracting the day at first infection from the day at final infection provided a measure of the duration of infection for each exposed fish. Because females are the more gregarious sex, we also analysed whether females contracted infections sooner than males. We took into account the unequal sex ratio, and compared the first day of infection of all secondarily exposed female and male guppies. We also calculated the average number of parasites on a fish, resulting in a measure of mean intensity during the duration of an infection. We further developed a measure of the success of the worm suprapopulation in each tank. This was simply the average daily number of worms present in a tank during the 90-day period of our experiment (tanks with shorter durations of infections were assumed to have remained uninfected from the last observation until the 90^th^ day).

We used a multivariate logistic regression to test whether the host-density treatment, the observed duration or intensity of infection (and their product, worm days) in the primary infected fish explained whether an epidemic occurred (defined as at least two new fish infected by at least four new worms). For tanks where transmission occurred, we used multivariate general linear models to explain how fish density, sex and whether or not the fish was the primary infected fish, affected the variation in worm days of each fish. To assess whether shoaling interactions affected transmission rates, we compared the day at first infection for males and females for the two tanks with 24 fish (these tanks had sufficient sample sizes for such a comparison). We also used a general linear model to determine whether host density affected the abundance of worms in a tank (parasite suprapopulations). For analyses that used fish as units of replication, tank was a random effect. All potential independent variables and their potential first-order interactions were first entered into an initial model. Final model selection was based on minimizing AIC. Variables were transformed, if necessary, so that residuals were normally distributed. All analyses were performed in JMP 7 software.

## Results

**Figure 1:**
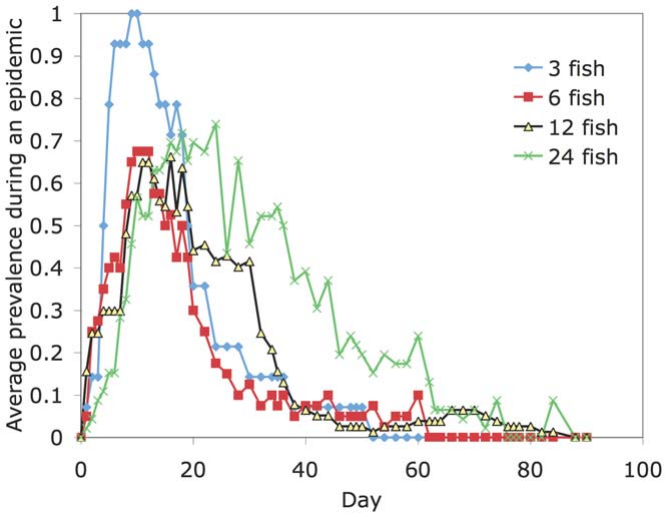
Proportion of infected fish (excluding the primary infected) over time in tanks where an epidemic occurred. Plotted is the average course of an epidemic (by density treatment).

**Figure 2:**
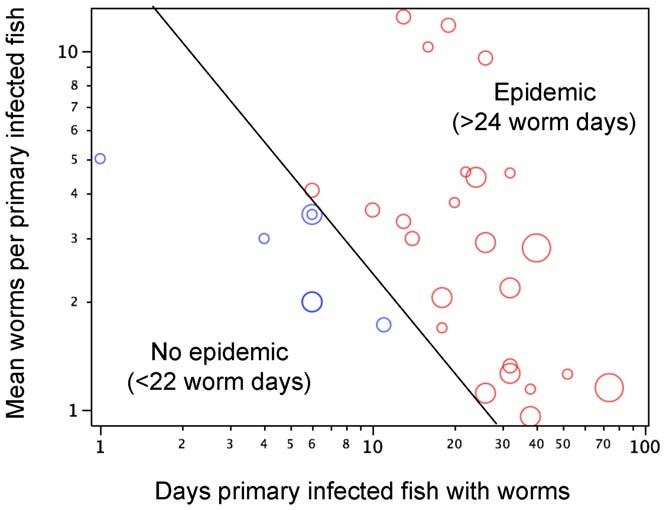
Characteristics of 24 tanks with an epidemic (transmission occurred) and seven tanks without an epidemic (no transmission occurred). Worm intensity (mean number of worms on a primary infected fish for the duration of infection) vs. the duration of infection (final day of infection minus initial day of infection). Circle size indicates the density of the treatment. The line (drawn to help visualization) divides tanks where one or more exposed fish became infected (transmission) from tanks where exposed fish remained uninfected (no epidemic).

**Figure 3:**
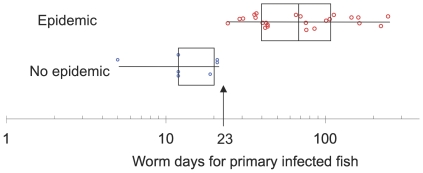
Epidemics and worm days for a primary infected fish. The box plot shows worm days per primary infected fish for epidemics and non epidemics. Boxes show quantiles, while whiskers show ranges. Points are individual tanks and are jittered on the vertical axis to reduce overlap. The figure is a simplified version of the data in Figure 2.

When transmission occurred, it was within the first week and the parasite suprapopulation persisted for an average of 62 days. Transmission to a single fish always resulted in an epidemic in the tank, and epidemics occurred with approximately the same probability irrespective of host density (i.e. in seven of the ten three-fish tanks, eight of the nine six-fish tanks, seven of the ten 12-fish tanks and both 24-fish tanks). Infections peaked rapidly after a couple of weeks and then faded, presumably as fish acquired immunity (Figure 1). If the primary infected fish failed to transmit its parasites, the parasite suprapopulation went extinct within 4–7 days. Except for two cases where the primary infected fish died late in an epidemic, the loss of infection in the primary infected fish appeared to be due to worm death or transfer. The logistic regression suggested that the number of worm days (Log(10) transformed) (Chi-sq. = 33.1, d.f. = 1, P<0.0001) experienced by the primary infected fish affected the probability of transmission to the secondarily exposed fish, but that fish density was not a significant effect (and so was dropped from the model) (Figure 2 shows how epidemics responded to duration, intensity and density). In tanks without epidemics, the primary infected fish was infected with an average of 2.8 (+/−0.46 s.e.) worms for 5.7 (+/−1.1 s.e.) days while in tanks where epidemics occurred the primary infected fish was infected with an average of 4.7 (+/−1.0 s.e.) worms for 28.6 (+/−3.3 s.e.) days (Figure 2). Perhaps more instructive is the non-overlap in the distribution of worm days between epidemics and non epidemics. In all tanks where epidemics occurred, the primary infected fish experienced 24 or more worm days, while, in all tanks without epidemics, the primary infected fish experienced 22 or fewer worm days (Figure 3). Fish density did not affect the worm days on the primary infected fish (general linear model, R-square = 0.006, F_1,29_ = 0.16, P = 0.69).

Transmission appeared to be related to social interactions in the tanks with 24 fish. Females, which shoal more, were the first two or three fish to become infected, but this could be (partly) explained by the 2∶1 female to male sex ratio. We therefore compared the first day of infection of all secondarily exposed female and male guppies, a measure that is independent of sex ratio. This showed that females were infected on average earlier (Day 8.2, s.e. = 0.88) than males (Day 12.0, s.e. = 0.96, no tank effect, P = 0.006).

**Figure 4:**
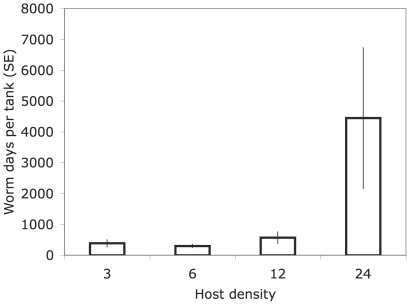
Mean daily suprapopulation in each density treatment. The suprapopulation is the average number of worms per tank per day for the duration of the experiment. The 24-fish treatment differed from the other treatments.

For tanks where an epidemic occurred, the worm days per fish was not significantly associated with fish sex, primary vs. secondary infected fish, or fish density. The maximum parasite intensity occurred in the highest density treatment 26 days post-infection, which was much later than in the lower density treatments (3-fish and 6-fish), where it occurred around Day 9 after first inoculation. However, this was probably related to the time it took for all fish within a treatment to become infected; not surprisingly, this was much longer for the higher host densities (see Table 1; Figure 4).

Intensity did not differ between secondarily exposed fish and primary infected fish, or between males and females. Intensity was highest in the lowest density treatment, and second highest in the highest density treatment (leading to no clear linear effect of density on intensity). In tanks with epidemics, worm suprapopulations increased with fish density as measured by average worms per tank per day (Log transformed, R-square = 0.34, slope = 0.04 (0.01 s.e.), F_1,22_ = 11, P = 0.003) or total worm days per tank (Log transformed, R-square = 0.41, slope = 0.05 (0.01 SE), F_1,22_ = 15.5, P = 0.0007), primarily due to the large number of worms in the tanks with 24 fish (Figure 3).

On average, the fish that died, died on day 33 (SD = 19, N = 56). The prevalence of infection for dead fish was 60%. Surprisingly, host mortality was not lower amongst the control fish; 21.3% died during the experiment, compared to 17.5% (252 fish) in treatment tanks (Chi-sq. = 0.579, d.f. = 1, P = 0.4467). This unusually high mortality rate in the controls suggests there was another unidentified cause of mortality in these tanks. It does not suggest, however, that the worms had little pathogenic effect on the fish, because amongst the infected fish, those with high worm intensities were more likely to die during the experiment (mean intensity for dead fish = 15.6, mean intensity for surviving fish = 2.7, t = −6.8, P = 0.0001). Similarly, tank mortality rate was positively associated with mean intensity (Log transformed, R-square = 0.30, slope = 0.07 (0.02 s.e.), F_1,29_ = 12.6, P = 0.001), but not guppy density.

## Discussion

In this experiment, we infected a guppy population with a gyrodactylid ectoparasite to examine how the density of the host population (3, 6, 12 and 24 fish/40 L) affected the epidemic. The four main results were: (1) fish density did not significantly affect the probability of an epidemic (2) the probability of an epidemic increased with the product of duration and mean intensity of infection in the primary infected fish, (3) female guppies were infected earlier in the epidemic than males, and (4) not surprisingly, the total parasite population increased with the host population density These findings are most consistent with frequency (rather than density) dependent transmission across the range of densities in our experiment. They can be explained by the fact that guppies are social, such that even at the lowest density in the current study, the contact rates among hosts were sufficiently high to allow transmission and higher densities did not increase contact rates enough to increase the probability of an epidemic [18]–[19], [28]. The importance of social contacts is underscored by the observation that female guppies contracted infections earlier than males [see also 35]. A higher contact rate for females is consistent with their more pronounced shoaling behaviour in comparison to males [20].

The transmission success increased significantly with the worm days experienced by the primary infected fish. Given that all primary infected fish were initially infected with four gyrodactylids at the start of the infection, important variation in severity of the epidemics stems from differences in reproduction and mortality rates of the parasite on the primary infected fish. This finding is consistent with models that consider how some hosts can be super spreaders, transmitting infection to large numbers of conspecifics [17], and suggests that the reproductive rate of a parasite on its host is an important but under-studied aspect of microparasite epidemics [36]. We would expect that transmission success should vary among primary infected fish simply due to the chance loss of worms during transfer. Past studies have shown that 40% of worms can be lost when attempting to transfer from one host to another [37]. However, this cannot explain all the variation in worm days among the primary infected fish because a simple calculation of the binomial distribution for successes and failures suggests that only 3% of fish infected with four worms would fail to transmit a single worm due to loss during contacts, which does not explain our 23% transmission failure. Therefore, it seems plausible that variation in tolerance or resistance [e.g. 26] among the primary infected fish, or loss in fitness due to inbreeding depression in the *Gt3* parasite strain [27], could have also influenced worm days in the primary infected fish. To conclude, characteristics of the primary infected fish appeared to influence epidemics more than characteristics of the susceptible population, but we do not know the source of variation in worm days among the primary infected fish.

This is the first study on gyrodactylids to monitor infection trajectories on groups of individually marked fish in controlled laboratory conditions (although see [38] for an experiment on *G. turnbulli* infection-dynamics on unmarked guppies). Our experiment was designed to evaluate basic epidemiological predictions, and the patterns we observed may not reflect how *G. turnbulli* epidemics proceed in nature. In particular, parasite intensities are relatively low in the wild [39]–[40]. This may be because wild guppies mix among shoals, which may comprise individuals with differing histories of exposure (and immunity). Furthermore, predators may differentially take sick fish, and scavengers may feed on carcasses, and the most heavily infected fish may be washed downstream [29] removing an important source of infection (in our tanks, worms from dead guppies were available to re-infect new hosts until we removed the dead fish).

Although our data are most consistent with frequency- rather than with density-dependent transmission, this does not imply that host density is unimportant in gyrodactylid infection dynamics. First, it is possible that transmission would be reduced at densities lower than the three fish per tank used in our experiment. Future experiments using larger tanks could reduce the number of fish per litre even lower than we were able to. Second, with only 31 replicates and a high frequency of epidemics, we had limited power to detect a subtle effect of density (e.g., both of the highest density treatments experienced an epidemic, but low replication for this treatment made it impossible to assign a clear density effect). Third, there may have been conflicting effects among the variables, making it difficult to conclude that contact rates were not affected by density. For instance, if the intimacy of interactions decreased with density, this could have cancelled an increase in the number of contacts with density. In any case, the lack of an association between epidemics and host density does not mean that host density is unimportant for the parasite population. In the highest density treatment, the parasite suprapopulation persisted longer and mean daily intensity was relatively high, leading to a substantially larger worm suprapopulation. The parasite suprapopulation probably benefited from a high host density due to the greater resource base and the longer period during which non-immune fish were available, suggesting bottom-up production for the parasite, as seen in many other consumer–resource interactions [41].

In addition to indicating how parasites can invade a host population, our results provide insight into the loss of parasites from host populations. Frequency-dependent transmission increases the likelihood of parasite persistence at low host densities [5], but it also increases the chance that a parasite can drive its host population extinct [6]. It is therefore noteworthy that several relatively isolated guppy populations in Trinidad appear to be completely free of this group of parasites [42], and the fish in these populations are extremely susceptible to gyrodactylid infections [27]. Furthermore, there are many suitable guppy habitats in upland rivers of Trinidad without guppies. This suggests that either the parasite and/or the host have not invaded into these habitats, or that in such relatively isolated locations, the parasite can drive itself and the host population to extinction.
